# Eratum

**DOI:** 10.1111/jdi.12979

**Published:** 2018-12-03

The Publisher would like to draw the reader's attention to the errors in the following article.

Haneda M, Noda M, Origasa H, Noto H, Yabe D, Fujita Y, Goto A, Kondo T, Araki E. Japanese Clinical Practice Guideline for Diabetes 2016. *J Diabetes Investig* 2018; 9: 657–697.

On page 657, the DOI mentioned in the footnote as a jointly published article in Diabetology International should have been https://doi.org/10.1007/s13340-018-0345-3. Also, ethics policy should have been stated as follows.

Ethics policy: The article does not contain any studies with human or animal subjects performed by any of the authors.

Disclosure should have been stated as follows.

## Disclosure

MH received honoraria for lectures for Astellas Pharma Inc., Taisho Toyama Pharmaceutical Co., Ltd., Mitsubishi Tanabe Pharma Corporation, Boehringer Ingelheim Japan, Inc., Taisho Pharmaceutical Co., Ltd., Kowa Pharmaceutical Co., Ltd., Ono Pharmaceutical Co., Ltd., MSD K.K., Novartis Pharma K.K., Novo Nordisk Pharma Ltd., and Sanofi K.K. and scholarship grants from Astellas Pharma Inc., Daiichi‐Sankyo Co., Ltd., MSD K.K., Mitsubishi Tanabe Pharma Corporation, Takeda Pharmaceutical Co., Ltd., Taisho Toyama Pharmaceutical Co., Ltd., Novo Nordisk Pharma Ltd., Elli‐Lilly Japan K.K., Boehringer Ingelheim Japan, Inc., Kyowa Hakko Kirin Co. Ltd., Ono Pharmaceutical Co., Ltd., Kowa Pharmaceutical Co., Ltd., Sanofi K.K., Shionogi & Co., Ltd., Johnson & Johnson K.K., Otsuka Pharmaceutical Co., Ltd., and Kissei Pharmaceutical Co., Ltd. MN received honoraria for lectures for MSD K.K. and scholarship grants from Mitsubishi Tanabe Pharma Corporation, Daiichi Sankyo Co., Ltd., MSD K.K., Novo Nordisk Pharma Ltd., Takeda Pharmaceutical Co., Ltd., Astellas Pharma Inc., Kyowa Hakko Kirin Co., Ltd., Boehringer Ingelheim International GmbH, and Sumitomo Dainippon Pharma Co., Ltd. HN received honoraria for lectures for Elli Lilly Japan K.K. DY received honoraria for lectures for Novo Nordisk Pharma Ltd., Boehringer Ingelheim Japan, Inc. and research grants from Terumo Corporation and scholarship grants from Taisho Toyama Pharmaceutical Co., Ltd., MSD K.K., Ono Pharmaceutical Co. Ltd., Novo Nordisk Pharma Ltd., Arklay Inc., and Takeda Pharmaceutical Co. Ltd. EA received honoraria for lectures for MSD K.K., Ono Pharmaceutical Co., Ltd., Sanofi K.K., Novo Nordisk Inc. and scholarship grants from Astellas Pharma Inc., Novo Nordisk Inc., Pfizer Japan Inc, Ono Pharmaceutical Co., Ltd., Sumitomo Dainippon Pharma Co., Ltd., and Sanofi K.K. HO, YF, AG, and TK declare no conflict of interest.

The Publisher apologizes for these errors and any confusion they may have caused.

